# Comparative evolutionary genomics of the HADH2 gene encoding Aβ-binding alcohol dehydrogenase/17β-hydroxysteroid dehydrogenase type 10 (ABAD/HSD10)

**DOI:** 10.1186/1471-2164-7-202

**Published:** 2006-08-09

**Authors:** Alexandra T Marques, Agostinho Antunes, Pedro A Fernandes, Maria J Ramos

**Affiliations:** 1 REQUIMTE, Departamento de Química, Faculdade de Ciências, Universidade do Porto, Rua do Campo Alegre, 687, 4169-007 Porto, Portugal

## Abstract

**Background:**

The Aβ-binding alcohol dehydrogenase/17β-hydroxysteroid dehydrogenase type 10 (ABAD/HSD10) is an enzyme involved in pivotal metabolic processes and in the mitochondrial dysfunction seen in the Alzheimer's disease. Here we use comparative genomic analyses to study the evolution of the HADH2 gene encoding ABAD/HSD10 across several eukaryotic species.

**Results:**

Both vertebrate and nematode HADH2 genes showed a six-exon/five-intron organization while those of the insects had a reduced and varied number of exons (two to three). Eutherian mammal HADH2 genes revealed some highly conserved noncoding regions, which may indicate the presence of functional elements, namely in the upstream region about 1 kb of the transcription start site and in the first part of intron 1. These regions were also conserved between Tetraodon and Fugu fishes. We identified a conserved alternative splicing event between human and dog, which have a nine amino acid deletion, causing the removal of the strand β_F_. This strand is one of the seven strands that compose the core β-sheet of the Rossman fold dinucleotide-binding motif characteristic of the short chain dehydrogenase/reductase (SDR) family members. However, the fact that the substrate binding cleft residues are retained and the existence of a shared variant between human and dog suggest that it might be functional. Molecular adaptation analyses across eutherian mammal orthologues revealed the existence of sites under positive selection, some of which being localized in the substrate-binding cleft and in the insertion 1 region on loop D (an important region for the Aβ-binding to the enzyme). Interestingly, a higher than expected number of nonsynonymous substitutions were observed between human/chimpanzee and orangutan, with six out of the seven amino acid replacements being under molecular adaptation (including three in loop D and one in the substrate binding loop).

**Conclusion:**

Our study revealed that HADH2 genes maintained a reasonable conserved organization across a large evolutionary distance. The conserved noncoding regions identified among mammals and between pufferfishes, the evidence of an alternative splicing variant conserved between human and dog, and the detection of positive selection across eutherian mammals, may be of importance for further research on ABAD/HSD10 function and its implication in the Alzheimer's disease.

## Background

The enzyme Aβ-binding alcohol dehydrogenase/17β-hydroxysteroid dehydrogenase type 10 (ABAD/HSD10) belongs to short chain dehydrogenase/reductase (SDR) family and its distinct features include the capacity to bind amyloid-beta peptide (Aβ) [1] and the ability to use a broad array of substrates, encompassing 3-hydroxyacyl-CoA derivatives, steroids, alcohols, and β-hydroxybutyrate [2-6]. ABAD/HSD10 has function in the mitochondria and is expressed in several tissues, including brain, liver and gonads [5]. The broad expression pattern together with the multiple substrate specificities enables the enzyme to participate in several metabolic processes (reviewed in [7]), namely, the oxidation of fatty acids and branched-chain amino acids [6,8], sex steroids metabolism in gonads [9,10] and oxidation of steroid modulators of GABA_A _receptors in brain [11].

ABAD/HSD10 was found to bind Aβ in a yeast-two hybrid screen against human brain and HeLa cDNA libraries [1]. Subsequently, various studies [12,13] provided evidence that ABAD/HSD10 can mediate the cytotoxic effects of Aβ in the mitochondrial compartment, thus contributing to the mitochondrial dysfunction seen in Alzheimer's disease (AD) (reviewed in [14]). The structure determination of human ABAD/HSD10 in complex with Aβ [12] revealed that the enzyme's loop D is the binding site of Aβ, and that binding of Aβ to ABAD/HSD10 leads to distortion of the ABAD/HSD10 structure, possibly inhibiting its enzymatic activity. Transgenic mice overexpressing ABAD/HSD10 in an Aβ-rich environment displayed neuronal oxidative stress, cell death and accelerated decline in spatial learning and memory [12,13]. In addition, ABAD/HSD10 expression was reported to be enhanced in brains from patients with AD when compared with brains from non-demented age-matched controls [12,13].

Although ABAD/HSD10 is an enzyme with function in the mitochondria it is encoded by a nuclear gene termed HADH2 (official name). The human HADH2 gene, mapped at chromosome Xp11.2, consists of six exons and five introns [8] and encodes a protein with 261 amino acids. The three-dimensional structure of ABAD/HSD10 has been determined with great resolution [2,15]. The enzyme has a homotetrameric structure, with each subunit containing a Rossman fold dinucleotide-binding motif, composed of a core β-sheet of seven parallel strands flanked by six α-helices, which is involved in the interaction with the cofactor (NAD). The C-terminal portion of the enzyme is involved in substrate binding and harbors the Ser/Lys/Tyr catalytic triad characteristic of the SDR family members [16].

Here we performed a thorough comparative genomic analysis of HADH2 genes from 21 organisms, including several species of mammals, amphibians, fishes, insects and nematodes. We provide insights into the evolution of such HADH2 genes, namely its genomic organization, patterns of sequence conservation, alternative splicing variants and phylogenetic relationships. Moreover, we provide evidence suggesting signatures of positive selection across eutherian mammal ABAD/HSD10 proteins, which may be of importance for more applied biomedical research on enzyme function and its implication in the Alzheimer's disease.

## Results

### Cross-species comparison of HADH2 gene organization

**Figure 1:**
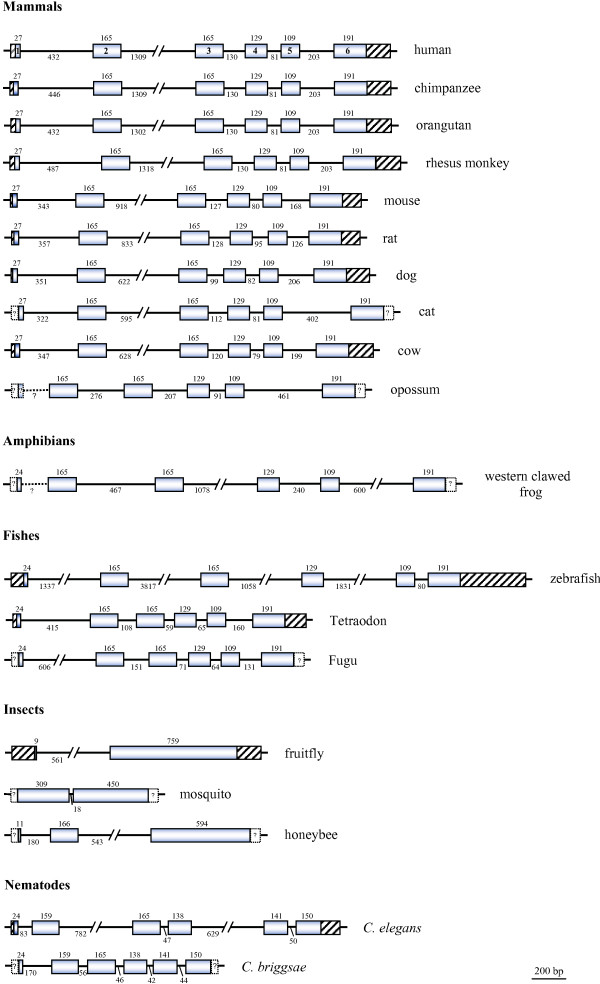
**Genomic organization of HADH2 genes**. Exons are represented by boxes and introns by lines; all are shown to scale (bar = 200 bp) except for intronic regions larger than 500 bp that are represented as slashed lines. Known 5' and 3' untranslated sequences are represented by boxes with black streaks. Exon sizes in base pairs are indicated above the boxes and intron sizes below the horizontal lines. All the genes are represented in the 5' → 3' orientation, with the exons being numbered in the human gene. Unknown regions of genes are represented by unscaled hatched lines and boxes with a question mark.

Vertebrate HADH2 genes showed a six-exon/five-intron organization, with the exon lengths and the localization of the exon/intron boundaries being highly conserved (Figure 1). The nematode genes also exhibited a similar gene organization, but the exon/intron boundaries involving the last three exons were in a different position relatively to the vertebrate orthologues and exon 2 contained a six nucleotide (two codons) deletion. Insects showed, however, a distinct HADH2 gene organization with a reduced and varied number of exons: two in the mosquito and fruitfly, and three in the honeybee. The length of the coding sequences was similar across the species analysed, ranging from 765 (fruitfly and mosquito) to 783 bp (mammals). In general, HADH2 genes were less than 3.20 Kb long (Table 1; Figure 1), with most of its introns shorter than 500 bp. The zebrafish gene was, however, much larger (9.38 Kb), which resulted from its four larger introns (ranging from 1.058 to 3.818 bp; Figure 1). A high proportion of repeats in the zebrafish intron 2 accounted for its notably larger size (see Additional file 1), but few or no repeats were found in the other introns. In eutherian mammals, HADH2 genes were very similar in size (2.16–3.17 Kb, Table 1). The main differences were related with the different accumulation of repeats in introns (see Additional file 1, Figure 1). SINEs of different sub-families occupied a moderate percentage of intron 2 in primates and rodents and of intron 5 in cat. Among invertebrates, *C. elegans *had the largest HADH2 gene. This is greatly related with the larger size of *C. elegans *introns 2 and 4, apparently resulting from an accumulation of DNA transposons (see Additional file 1).

**Table 1:** Accsession numbers, chromosome location and GC content of HADH2 genes

**Species**	**Accession Number**	**Chromosome location**	**Gene length (Kb)**	**GC**_**gene**_	**GC**_**coding**_		
					
					**GC**_**1**_	**GC**_**2**_	**GC**_**3**_
human (*Homo sapiens*)	ENSG00000072506	chromosome X (p11.2)	3.11	52	66	46	63
chimpanzee (*Pan troglodytes*)	ENSPTRG00000021929	chromosome X	3.12	52	66	46	63
orangutan (*Pongo Pygmaeus*)	^a^	-	3.11	52	66	45	63
rhesus monkey (*Macaca mulatta*)	ENSMMUG00000009296	geneScaffold_500	3.17	51	66	45	65
rat (*Rattus norvegicus*)	ENSRNOG00000003049	chromosome X	2.45	48	64	44	52
mouse (*Mus musculus*)	ENSMUSG00000025260	chromosome X	2.56	49	64	44	50
dog (*Canis familiaris*)^b^	ENSCAFG00000016277	chromosome X	2.29	52	66	44	61
cat (*Felis catus*)^c^	^a^	-	2.30	51	66	44	60
cow (*Bos Taurus*)	ENSBTAG00000017779	chromosome X	2.32	52	64	46	62
pig (*Sus scrofa*)^d^	TC220713	-	-	-	66	45	66
opossum (*Monodelphis domestica*)^e^	ENSMODG00000020889	scaffold_255	1.80	55	66	46	64
western clawed frog (*Xenopus tropicalis*)^f^	ENSXETG00000007721/TC2334	scaffold_154	3.17	44	63	43	48
african clawed frog (*Xenopus laevis*)^d^	TC274805	-	-	-	62	44	48
zebrafish (*Danio rerio*)	ENSDARG00000017781	chromosome 18	9.38	33	62	47	49
Fugu (*Takifugu rubripes*)^c^	SINFRUG00000123807	scaffold_2159	1.81	51	66	47	69
Tetraodon (*Tetraodon nigroviridis*)	GSTENG00023338001	chromosome 9	1.73	57	67	48	81
fruitfly (*Drosophila melanogaster*)	CG7113	chromosome X	1.61	51	62	44	83
mosquito (*Anopheles gambiae*)^c^	ENSANGG00000012647	chromosome 2R	0.77	60	67	42	73
honeybee (*Apis mellifera*)	ENSAPMG00000015843	group11	1.49	18	54	38	12
*Caenorhabditis elegans*	F01G4.2	chromosome IV	2.50	34	63	43	35
*Caenorhabditis briggsae*^c^	CBG06017	assembly cb25.fpc0143	1.13	43	61	43	40

^a ^These gene sequences were manually inferred based on species specific WGS sequences retrieved from a tblastn search to NCBI trace archive: orangutan – ti812046525, ti812049734, ti848517349, ti718725185; cat – ti651599423, ti653340876, ti825822144, ti646417680, ti818302482, ti826254688

^b ^The gene sequence does not include the 5' UTR sequence

^c ^The gene sequences does not include the UTR sequences

^d ^No information available about intronic sequences

^e ^The gene sequence does not include the first exon, first intron and UTRs sequences

^f ^The gene sequence does not include the first intron and UTR sequences

The gene overall GC content was similar among the eutherian mammals (48–55%), but substantially heterogeneous among fishes (33–57%), insects (18–60%) and nematodes (34–43%). The GC content of third-codon position (GC3) showed a high heterogeneity across HADH2 genes, while GC content in the first (GC1) and second-codon (GC2) positions were more homogeneous. Previously, it was reported that in most vertebrate genomes, GC3 levels are correlated with the GC level of the isochore region containing the gene [17,18]. Accordingly, the vertebrate HADH2 genes with high GC3 values should probably be localized in GC-rich isochores, which also preferentially allow the accumulation of SINE repeats. Conversely, the comparatively low GC3 values observed in rodents, amphibians and zebrafish HADH2 genes should probably reflect a shift toward GC-poorer regions. Interestingly, honeybee HADH2 gene was remarkably AT-biased, which was related both to the very low GC3 content and the extreme predominance of AT-rich repeats in its introns (Additional file 1).

Eutherian mammals HADH2 genes were localized in the X chromosome (Table 1), confirming the known high synteny conservation of eutherian X chromosomes [19]. Tetraodon HADH2 gene was localized on chromosome 9, which was previously reported to show a high synteny with human Xp11.2 region [20]. Zebrafish HADH2 gene was, however, localized on chromosome 23, which has a very low synteny either with human chromosome X or with Tetraodon chromosome 9 [21].

**Figure 2:**
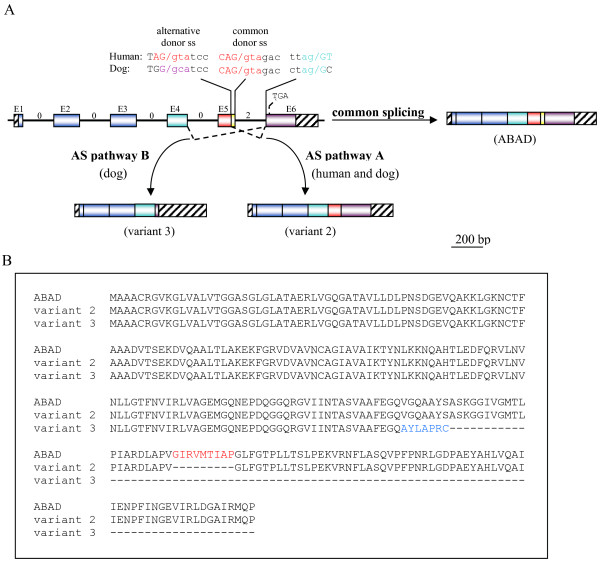
**Alternative splicing events identified in the human and dog HADH2 genes**. **A**) Schematic illustration of the splicing pathways of human and dog HADH2 genes. The typical exon-intron organization of human and dog HADH2 genes is shown. Exons (E), which are of same lengths in human and dog genes, are represented by coloured boxes and shown to scale (bar = 200 bp). Introns and UTR regions are schematized by unscaled lines and boxes with black streaks, respectively. Intron phases are indicated by numbers above the lines. Alternative splicing (AS) pathway A, identified in human and dog, involves utilization of an alternative donor splice site (ss) in the exon 5, resulting in a nine amino acid deletion. The sequences of human and dog intron 5 splice sites (ss) and of the alternative donor splice site are shown. The conserved nucleotides that identify the U2-type consensus sequences for the GT splice donor site (MAG**GT**RAGT), GC splice donor site (MAG**GC**AAGT) and acceptor splice site (NC**AG**GT) are in red, violet, and green. Alternative splicing (AS) pathway B, identified in dog, involves the skipping of exon 5 which, due to the different phase of introns 4 and 5, leads to the appearance of an earlier stop codon (TGA) in the exon 6, resulting in the replacement of the last 92 amino acids by seven new ones. **B**) Amino acid sequences of the dog ABAD/HSD10 and variants produced by AS. The nine amino acids deleted in the variant 2 (252 amino acids) are in red (The nine amino acids deleted in the human variant 2 homologue are identical). The seven new amino acids in the variant 3 are in blue.

**Figure 3:**
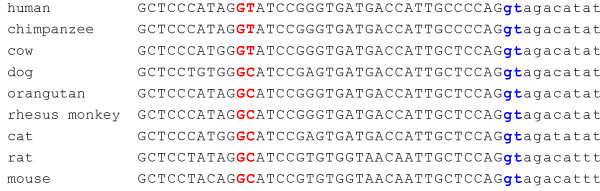
**Conservation of the human and dog alternative donor splice site in the exon 5 of the other eutherian mammals**. The last 30 nucleotides (in uppercase) of exon 5 and the first 10 nucleotides (in lowercase) of intron 5 are given. The canonical GT dinucleotides in the common donor splice site of intron 5 are in blue while the canonical GT and non-canonical GC dinucleotides in the alternative donor splice site are in red.

**Figure 4:**
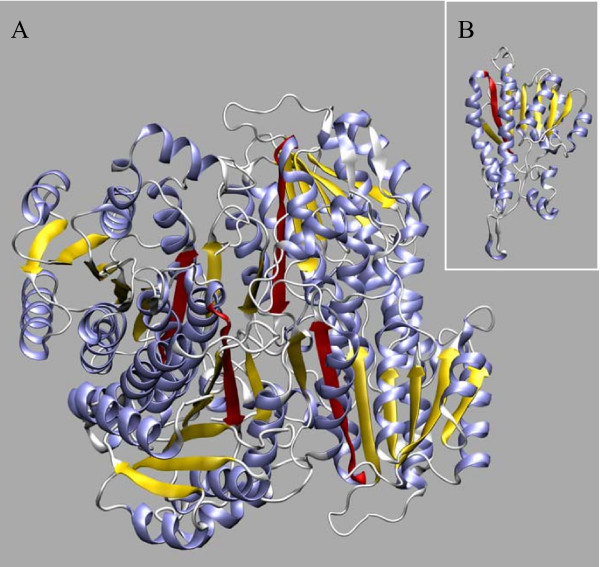
**Localization of the strand β_F _deleted in the human and dog variant 2 produced by alternative splicing**. The strand β_F _is highlighted in red both in the human ABAD/HSD10 tetramer (A) and in the one of its monomers (B) (PDB code 1U7T). This strand together with other six (coloured yellow) comprise the core β-sheet of the Rossmann fold dinucleotide-binding motif. Helices are in blue and the other secondary structure elements are in white.

The evaluation of mRNA RefSeq databases at NCBI revealed the existence of an alternative spliced variant, with 252 amino acids (variant 2), both in human (NM_001037811) and dog (XN_859362). This variant is generated through the use of a different 5' splice site of intron 5, localized 27 nucleotides upstream of the normal donor splice site, resulting in a nine amino acids deletion (residues 190–198) without disrupting the reading frame (splicing pathway A in Figure 2). Such deletion causes the removal of strand β_F _(residues 191–198) which, together with other six strands, compose the core β-sheet of the Rossmann fold dinucleotide-binding motif (Figure 4). Typically, HADH2 introns from the species analysed contained the canonical GT and AG dinucleotides in the donor and acceptor splice sites, respectively. However, while the human alternative donor splice site contained a GT dinucleotide, a rare GC dinucleotide was present in the dog alternative donor splice site (Figure 2). The GC-AG splice sites are the major non-canonical splice sites, comprise 0.5–1% of mammalian splice sites and, like the GT-AG splice sites, are also processed by the standard U2-type spliceosome [22,23]. Both human and dog alternative donor splice sites match poorly the known mammalian splice-sites consensus sequences [24] than the normal intron 5 donor splice sites. The dog and human normal intron 5 donor splice sites match the mammalian U2-dependent consensus sequence for the GT splice site (MAGGTRAGT) in six out of nine residues while the human alternative 5'-splice site matches only five residues. Dog alternative donor splice site matches the consensus sequence for the GC splice site (MAGGCAAGT) only in four out of nine nucleotides (Figure 2). The poor match of alternative splice sites with splice-site consensus sequences is not uncommon. In fact, the alternative splicing is usually associated with weak splice signals, and variability in signal strength can act as an underlying regulatory mechanism [25,26]. The other eutherian mammal genes have highly conserved alternative donor splice sites with those of dog and human (Figure 3), suggesting that they might also potentially express a similar alternative splicing variant. In dog, we identified a second alternative splicing variant (XM_859344) with 169 amino acids (variant 3). It results from skipping exon 5, which causes a shift of the reading frame, leading to the appearance of a premature stop codon in the exon 6 (splicing pathway B in Figure 2). The last 92 amino acids are replaced by seven new ones, resulting in the loss of important functional residues, such as, two residues of the catalytic triad (Tyr168 and Lys172) and the residues (202–220) comprising the substrate binding loop.

### Patterns of nucleotide sequence conservation among HADH2 genes

**Figure 5:**
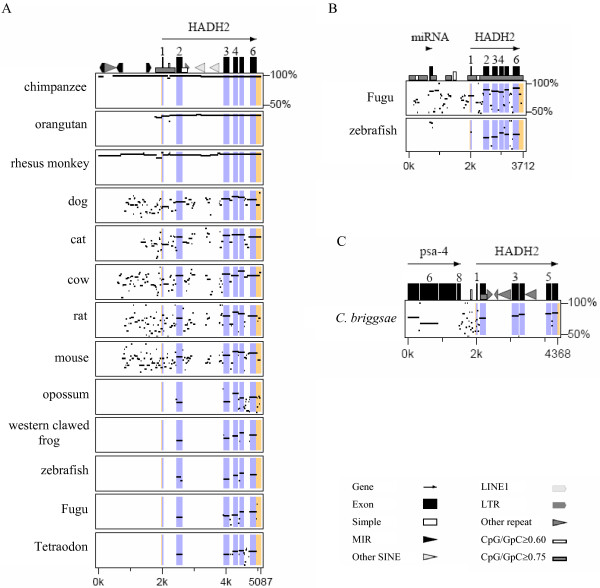
**Percent identity plots (PIPs) comparing the HADH2 gene and 5'-flanking sequences between human and the other vertebrates (A), Tetraodon and the other fishes (B), and *C. elegans *and *C. briggsae *(C)**. The reference sequences are scaled in kilobases. Percent sequence identity (50% – 100%) is shown on the *y*-axis. RepeatMasker was used to locate repeat elements. The gene orientation (5' → 3') is shown by the *arrow*. Exons are illustrated by vertical black boxes and highlighted blue across the plots; UTRs are highlighted yellow across the plots. For cat and orangutan, only the 1.2 kb and 200 bp 5'-flanking sequences were available. *LTR*, long terminal repeat elements; *MIR*, mammalian interspersed repeats; *SINE*, short interspersed nuclear elements.

The Multipipmaker analysis involving the comparisons between human and each of the other vertebrate HADH2 genes revealed a reasonable conservation of the coding regions, especially in the last three exons, while introns and upstream regions were less or not conserved (Figure 5A). The human and the other primate sequences aligned along almost their entire lengths, with the region corresponding to the HADH2 gene showing an identity above 96%. Few insertions were detected, with the largest ones being associated with repetitive DNA. As expected, due to an increase in phylogenetic distance, the human sequence was less conserved with those of non-primate eutherian mammals, especially within noncoding regions. Considering the intronic regions, the overall percent identity was 77.1% for human/dog, 78.8% for human/cat, 76.3% for human/cow, 71.3% for human/mouse and 68.9% for human/rat. The lower similarity between human and the rodent introns, despite mouse and rat being considered closer to humans than the other non-primate eutherian mammals [27], likely reflect the higher mutation rate in the mouse/rat lineage [28]. Conservation between human and the other vertebrate introns was restricted to small regions flanking exons.

The comparisons among fish (Figure 5B) revealed that conservation between pufferfishes and zebrafish sequences was largely limited to coding regions and to a microRNA (family let-7) positioned upstream of the HADH2 gene, reflecting the deep divergence of the Euteleostei (110–160 million years; Myr) [29]. Despite the much shorter evolutionary distance separating Fugu and Tetraodon (18–30 Myr) [30], the intron identity between the two pufferfishes (67.7%) was lower than the intron identity between human and each of the other non-primate eutherian mammals (> 70 Myr divergence) [31]. This might be related with the reported high neutral nucleotide substitution rate between Fugu and Tetraodon, which was shown to be greater than that between human and mouse [30]. *C. elegans *and *C. briggsae *HADH2 genes were substantially conserved in coding regions but not in the introns (Figure 5C), which is supported by previous inferences of the reduced conservation of noncoding sequences between the genomes of these nematodes, due to their large divergence (~100 Myr) [32]. Insect HADH2 genes were conserved only in HADH2 coding regions. The divergent organization of insect HADH2 genes and the extremely high content of AT-rich repeats in the honeybee introns ruled out any conservation among insect introns or upstream regions.

High conservation of noncoding sequences across multiple species may indicate the presence of functional elements. The Multipipmaker analysis (Figure 5) showed the existence of gap-free alignments in noncoding regions with more than 70% identity, conserved across all human/eutherian mammals and to a lesser extent between Tetraodon and Fugu. Within the HADH2 upstream region, the portion at about 1 kb of the transcription start site showed an increase in conservation, with the alignment from the positions 897 bp to 1239 bp (human sequence) exhibiting > 73% identity for human/eutherian mammals. The upstream regions of Tetraodon and Fugu also displayed highly conserved alignments, in particular one, positioned very close to the miRNA, with 88% identity along 126 bp. Considering intronic regions, the first part of intron 1 contained the largest (130 bp) gap-free alignment conserved (72–73% with the human sequence) between human, carnivores and cow, which also overlapped with some shorter human/rodent gap-free alignments with ≥70% identity. Interestingly, the largest conserved gap-free alignment (72% identity along 89 bp) between Tetraodon and Fugu introns was also in the beginning of pufferfishes intron 1. Among eutherian mammals, regions of high conservation were also detected in the other introns. In the smaller intron 4, the last 50 bp alignment showed an identity above 75% for all human/eutherian mammal comparisons. The intron 5, which is involved in an HADH2 alternative splicing event similar in human and dog, showed an identity above 70% in the last 69 bp for all human/eutherian mammal comparisons. The 3'UTR region, which is frequently highly conserved among orthologue genes due to its involvement in pos-transcriptional control, displayed an identity above 70% in the human/eutherian mammal comparisons.

### Phylogenetic analyses

**Figure 6:**
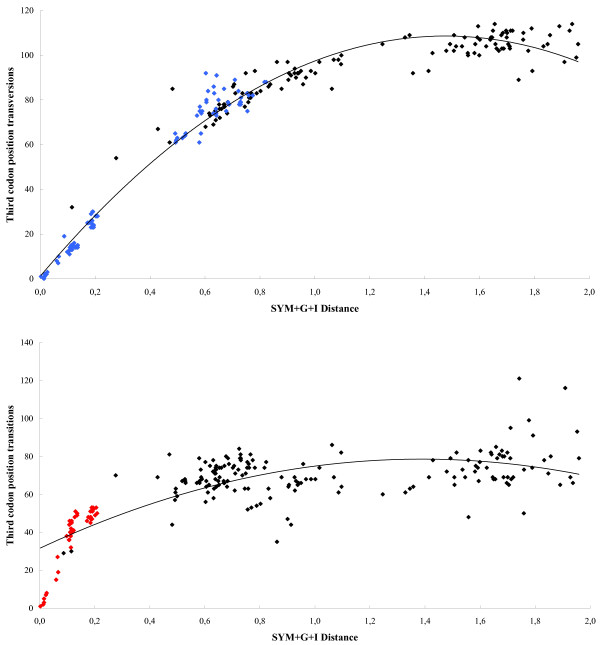
**Saturation plots of third codon position transitions and transversions versus corrected pairwise sequence divergence**. All comparisons between vertebrates and invertebrates are shown. The dots coloured blue in the first plot are for vertebrate pairwise comparisons, showing that third codon position transversions are not saturated for vertebrates, starting to saturate only for pairwise comparisons involving the invertebrate sequences. The dots coloured red in the second plot are for pairwise comparisons among eutherian mammals (the small saturation observed is due to some pairwise comparisons involving the mouse and rat sequences).

In the 783 bp alignment of the HADH2 coding region sequences, 549 nucleotides were variable and 489 were phylogenetically informative (PI). Considering PI sites, 134 (27.4%) were at first-codon position, 103 (21.1%) at second-codon position and 252 (51.5%) at third-codon position. However, as third-codon positions showed a significant base compositional bias and nucleotide saturation, they were excluded from phylogenetic analyses. Indeed, the saturation plots performed for each codon-position showed that third-codon position transitions and transversions start to saturate at different distances (Figure 6), while no significant saturation was observed at the first and second-codon positions (results not shown). It must be noted that the slight third-codon position transitions saturation observed for eutherian mammals (red dots in figure 6) is ascribed to the comparisons involving mouse and rat HADH2 sequences, which might be due to the recognized higher nucleotide substitution rate within these rodent species [28]. The base compositional bias at third-codon position reflected the extensive GC3 variation among species (Table 1), thus, accounting for 10 non-mammalian sequences rejecting the Tree-puzzle homogeneity chi-square test. By contrast, all sequences passed the chi-square test on second-codon positions while only honeybee sequence failed the test on first-codon positions. Recombination and gene conversion events, which can also interfere with phylogenetic analysis, were not detected across HADH2 genes.

**Figure 7:**
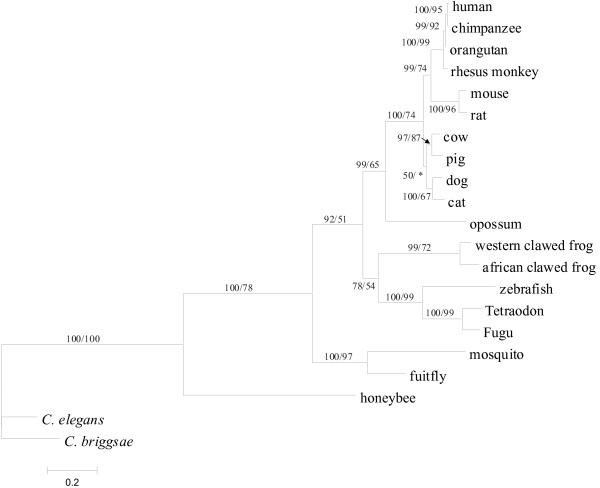
**Phylogenetic relationships of the 21 HADH2 genes analysed**. The Bayesian phylogenetic tree is shown (the ML tree produced an identical topology). Numbers above the branches are BPP and BS (based on 100 replicates) values (BPP/BS). The branch marked with (*) received a BS lower than 50%.

ML and Bayesian trees showed identical topologies (Figure 7). Both trees placed amphibians and fishes HADH2 genes in a monophyletic clade sister to the mammal clade, not reproducing the assumed topology placing amphibians closer to amniotes than fishes. The great divergence between honeybee and diptera (fruitfly and mosquito) HADH2 genes, as evidenced by their very dissimilar GC contents, might have precluded their placement in a single clade corresponding to insects. The phylogenetic relationships for mammal HADH2 genes are well within the known phylogeny of mammals [27], placing the rodents and primates as sister groups (one clade) and the artiodactyls (pig and cow) and carnivores (dog and cat) as sister groups in another clade. The clade containing artiodactyls and carnivores received, however, a low BPP and BS values (Figure 7). Nevertheless, it is remarkable to realize the high degree of congruence between the phylogeny of the HADH2 genes and the assumed eukaryotic phylogenetic tree, given the shorter size of the HADH2 sequences (522 bp, excluding third-codon position) and the great evolutionary divergence of the species analyzed (>1 billion years). Indeed, in general, reliable vertebrate and invertebrate phylogenetic trees are constructed based on larger data sets of concatenated genes because this allows increasing the number of PI sites (e.g. [27]).

### Patterns of ABAD/HSD10 amino acid sequence conservation

**Figure 8:**
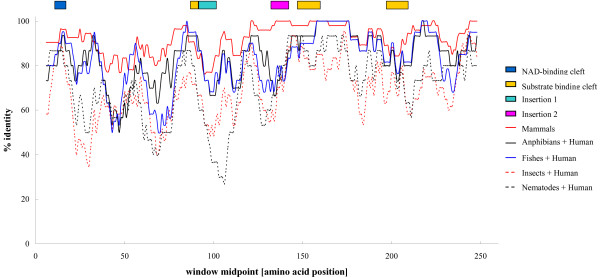
**Sliding window plots of the percentage of amino acid identity for comparisons between the human ABAD/HSD10 and the orthologue sequences within each group of species analysed**. The mammals plot does not include the opossum sequence as information about the amino acids encoded by the first exon was missing. The coloured bars at the top ilustrate the localizations of important functional regions of ABAD/HSD10. Window size was 10 bp and windows were moved in 1 bp steps.

ABAD/HSD10 amino acid sequences showed a high conservation among mammals (72–100%), amphibians (96%), fishes (86–95%), nematodes (95%), but slightly less among insects (63–82%). The ABAD/HSD10 amino acid identity between human and the other species (see Additional file 2) ranged from 59% (with nematodes) to 100% (with chimpanzee). Comparisons between human and each of the species group orthologues showed that the C-terminal region, where is localized most of the residues belonging to the substrate binding cleft and involved in subunit association [2,15], was clearly more conserved than the N-terminal region (Figure 8). The catalytic triad (Ser155, Tyr168 and Lys172), characteristic of the SDR family members, and the surrounding residues, were highly conserved. The substrate binding loop (residues 202–220), was found to be slightly less conserved. As in other SDR enzymes, the ABAD/HSD10 substrate binding loop is a mobile region, which may undergo conformational changes upon substrate binding [2]. A distinct feature of ABAD/HSD10 is the presence of two insertion regions (insertion 1 = residues 100–110; insertion 2 = residues 140–150), absent in the other SDRs. Insertion 1, which is localized in loop L_D _(residues 95–113), recently suggested to be the binding site for Aβ [12], exhibited a smaller degree of conservation relatively to insertion 2. Both insertions were also previously suggested to support the binding of CoA substrates [2]

### Adaptative selection on eutherian mammal ABAD/HSD10 proteins

At the gene-level approach, comparisons between site models M1a/M2a provided no significant evidence of selection in eutherian mammals. The model M8 fitted the data better than model M7, but the LRT comparing the two models was not significant (*P *> 0.05), with the BEB approach identifying 11 positively selected sites with posterior probabilities well below the 95% cutoff (Table 2). To test for variable ω ratios among lineages, the one ratio model was compared with the free ratio model. Although the free-ratio model fitted the data non significantly better than model M0 (*P *> 0.05, Table 2), it predicted variable ω values among branches, assigning a ω below 1 to nearly all branches, excepting to the branch separating human and chimpanzee from orangutan. The two-ratio model applied to this branch was significantly better than the model M0, indicating that the ω for the branch leading to the human and chimpanzee was significantly different than that found in the other eutherian branches of the tree.

**Table 2:** Results of the gene level approach (PAML) applied in eutherian mammal HADH2 genes

**Model**	***l***	**Parameter estimates**		**2Δ*l***	***P*-value**
**Site specific**					
Neutral (M1a)	-2436.668	*p*_0 _= 0.824, (*p*_1 _= 0.176)			
		ω_0 _= 0.035 (ω_1 _= 1)	M1a vs M2a	0	1
Selection (M2a)	-2436.668	*p*_0 _= 0.824, *p*_1 _= 0.114, (*p*_s _= 0.062)			
		ω_0 _= 0.035 (ω_1 _= 1) w_s _= 1			
Beta (M7)	-2444.085	*p *= 0.091, q = 0.371			
Beta&w (M8)	-2444.074	*p*_0 _= 0.919, (*p*_1 _= 0.106), q = 0.728	M7 vs M8	0.022	0.989
		*p*_s _= 0.08098, ω_s _= 1			
					
**Branch specific**					
One ratio (M0)	-2473.927	ω = 0.176			
Free-ratio	-2462.975		M0 vs free-ratio	22.420	0.146
Two-ratio	-2470.333	ω_0 _= 1.302 (foreg.)	M0 vs two-ratio	7.188	0.007*
		ω_1 _= 0.167 (back.)			

NOTE. – In M1 and M2 models, *p*_0_, *p*_1_, and *p*_s _are the proportions of codons estimated to each ω class. In M7 and M8 models, p and q are the parameters of the beta distribution and *p*_s _is the proportion of codon sites assigned to the positive selection class. M0 assumes a constant ω value while the free-ratio model assumes an independent ω ratio for each branch. Two-ratio model assumes a different ω ratio for the foreground branch (branch separating human and chimpanzee from orangutan) and background branches. 2Δ*l *is the log likelihood ratio statistic and *l *is the log likelihood value estimated for each model. No sites were identified under positive selection within the 95% confidence level by models M2a and M8; sites 5, 14, 49, 56, 57, 106, 59, 98, 103, 123, 239 were identified under model M8 with a *P *< 0.75.

**Figure 9:**
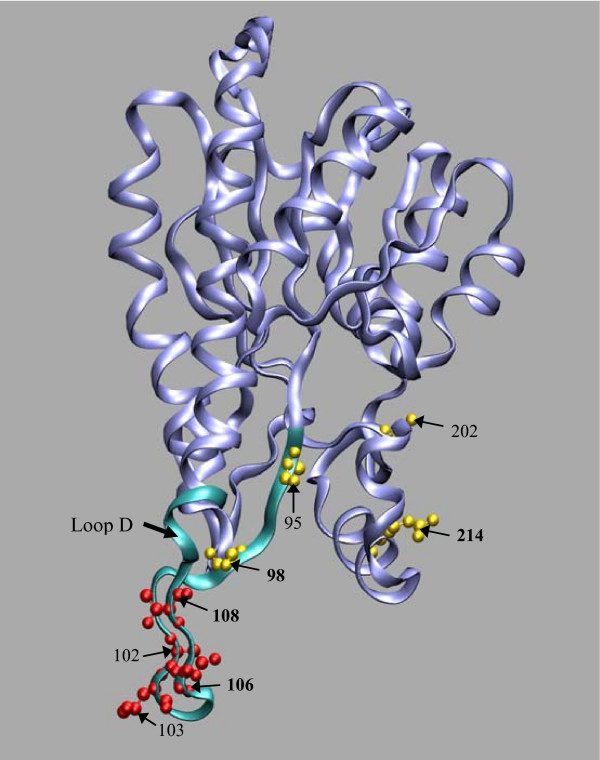
**Amino acid sites in important functional regions predicted to be under molecular adaptation by the protein-level approach**. The ribbon representation of a human ABAD/HSD10 monomer (PDB code 1U7T) is shown. The sites under molecular adaptation identified in the insertion 1 region and in regions comprising the substrate binding cleft are represented in the CPK form, coloured red and yellow, respectively. Sites in bold are the sites identified to be under positive selection in the branch separating human and chimpanzee from orangutan. The loop D region which is important for interaction of ABAD/HSD10 with Aβ is in green.

**Figure 10:**
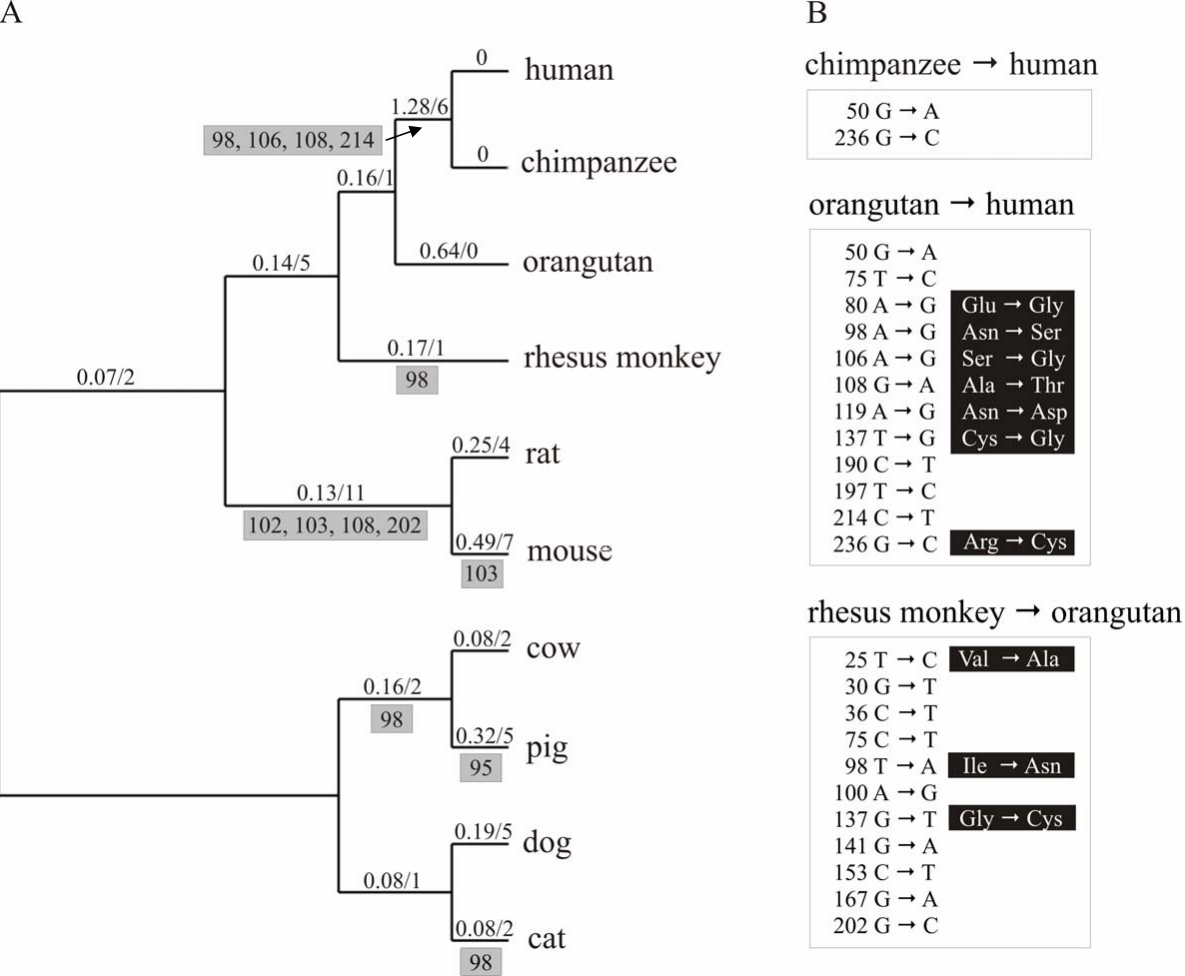
**Selective pressures across the different eutherian mammal lineages**. **A**) ML phylogeny of placental mammal HADH2 genes. The numbers above the branches are the ω ratio (left) estimated under the free-ratio model using PAML and the number of sites (right) that showed destabilizing selection among the physicochemical properties tested using TreeSAAP. A ω >1 suggests that positive selection has acted along that lineage; a ω = 0 reflects the absence of nonsynonymous substitutions. Below the branches, grey boxes list the sites showing destabilizing selection belonging to important functional regions of the protein (see table 3). The analysis was conducted using an unrooted topology (the topology is rooted for convenience). **B**) Nucleotide substitutions that occurred among great apes and between orangutan and rhesus monkey. The numbers refer to codon positions; for the nonsynonymous substitutions is listed the corresponding amino acid changes.

At the protein-level approach, we identified 36 ABAD/HSD10 amino acid sites showing properties under positive destabilizing selection among 28 of the 31 physicochemical properties tested (Table 3). Some of those sites were localized in particularly important functional regions. Six were localized in loop D: two (95 and 98) in a region belonging to the substrate binding site cleft and four (102, 103, 106 and 108) in the insertion 1 region (Figure 9). Two sites (202 and 214) belonging to the substrate binding loop were also identified to be under positive destabilizing selection. Among the 36 sites found to be under adaptative selection, some were detected in more than one branch of the tree, with the number of sites as well as the sites found in the referred important functional regions being listed for each eutherian branch in figure 10. Overall, positive destabilizing selection seems to have operated in several properties (e.g., properties P_α_, P_β_, N_s_, C_a_, α_c_, F, α_m _α_n_; see Table 3) related with the conformational and structural aspects of the enzyme. However, properties more likely to induce changes in the chemical environment (e.g., properties pK', pH_i_, P_r _and P and H_p_; see Table 3) were, overall, less affected by adaptative selection. Site 98, which was detected in a great number of branches (Figure 10), was the site containing the highest number of properties influenced by positive destabilizing selection (Table 3).

**Table 3:** Results of the protein-level approach (TreeSAAP) showing the amino acid sites and physicochemical amino acid properties influenced by positive-destabilizing selection among eutherian mammal ABAD/HSD10 proteins.

**Amino acid site**	**Properties**	**Amino acid site**	**Properties**
Substrate binding-cleft sites		56	*P*_α_, *α*_*m*_, *pH*_*i*_
95	*P*_α_, *pK'*	57	*N*_*s*_, *B*r, *μ*
**98**	*B*_*l*_, *R*_*F*_, *α*_*n*_, *R*_*a*_, *H*_*p*_, *pK'*, *H*, *N*_*s*_, *B*_*r*_, *μ*	59	*K*^*0*^, *M*_*w*_, *V*^*0*^, *M*_*w*_, *H*_*t*_
202	*P*_β_, *B*_*l*_, *K*^*0*^, *R*_*a*_	62	*K*^*0*^, *C*_*a*_, *M*_*w*_, *V*^*0*^
**214**	*pH*_*i*_, *F*, *P*, *α*_*c*_, *P*_*t*_	64	*α*_*c*_
Sites in insertion 1 region of loop D		70	*α*_*c*_
102	*N*_*s*_, *B*_*r*_, *E*_*l*_, *H*_*p*_, *E*_*t*_	73	*P*_α_
103	*B*_*l*_, *C*_*a*_, *H*, *Mw*, *V*^*0*^, *F*	76	*K*^*0*^
**106**	*P*_*c*_, *pK'*, *F*, *P*_*t*_	**80**	*P*_α_, *α*_*m*_
**108**	*P*_α_, *E*_*sm*_	**119**	*N*_*s*_, *P*_β_, *B*_*r*_, *H*_*p*_, *E*_*t*_
Other sites		123	*α*_*n*_, *R*_*a*_, *H*_*p*_
5	*C*, *R*_*a*_	135	*P*_*r*_, *P*, *α*_*m*_
7	*P*_*c*_, *pK'*, *F*, *P*_*t*_	177	*N*_*s*_, *P*_β_, *B*_*l*_, *R*_*F*_, *R*_*a*_, *H*_*p*_, *H*_*t*_
15	*α*_*c*_	189	*α*_*n*_, *R*_*a*_, *H*_*p*_, *α*_*c*_
25	*P*_α_, *E*_*sm*_	194	*α*_*c*_
28	*α*_*m*_	237	*K*^*0*^, *M*_*w*_, *V*^*0*^
44	*N*_*s*_, *B*_*r*_, *H*_*p*_, *E*_*t*_, *P*_β_	239	*E*_*sm*_, *P*_α_
46	*pK'*, *F*, *P*_*r*_, *α*_*c*_	240	*α*_*n*_
49	*E*_*sm*_, *P*_α_	244	*C*_*a*_, *M*_*w*_, *V*^*0*^
50	*F*		

Note. – Alpha-helical tendencies (*P*_α_), Average number of surrounding residues (*N*_s_), Beta-structure tendencies (*P*_*β*_), Bulkiness (*B*_*l*_), Buriedness (*B*_*r*_), Chromatographic index (*R*_*F*_), Coil tendencies (*P*_*c*_), Composition (*C*), Compressibility (*K*^*0*^), Equilibrium constant (ionization of COOH) (*pK'*), Power to be at the middle of alpha-helix (*a*_*m*_), Helical contact area (*C*_*a*_), Hydropathy (*H*), Isoelectric point (*pHi*), Long-range non-bonded energy (*E*_*l*_), Mean r.m.s. fluctuation displacement (*F*), Molecular volume (*M*_*v*_), Molecular weight (*M*_*w*_), Normalized consensus hydrophobicity (*H*_*nc*_), Partial specific volume (*V*^*0*^), Polar requirement (*P*_*r*_), Polarity (*P*), Power to be at the C-terminal (*α*_*c*_), Power to be at the N-terminal (*α*_*n*_), Refractive index (μ), Short and medium range non-bonded energy (*E*_*sm*_), Solvent accessible reduction ratio (*R*_*a*_), Surrounding hydrophobicity (*H*_*p*_), Thermodynamic transfer hydrohphobicity (*H*_*t*_), Total non-bonded energy (*E*_*t*_), Turn tendencies (*P*_*t*_). Residues in bold are those selected in the branch separating human and chimpanzee from orang-utan.

The evidence of positive selection, suggested both by the gene-level and the protein-level approaches in the lineage separating human/chimpanzee from orangutan reflects both the particular higher nonsynonymous than synonymous substitutions in that lineage and also the fact of most of the amino acid changes were nonconservative, thus likely to result in modifications of the physicochemical amino acid properties (Figure 10). Human/chimpanzee and orangutan differed in seven and five (three in case of chimpanzee) nonsynonymous and synonymous substitutions, respectively. By contrast, the other eutherian mammal branches revealed a considerably greater number of synonymous than nonsynonymous substitutions (results not shown). Among primates, human and chimpanzee proteins were identical while a higher number of amino acid differences was observed between human (and chimpanzee) and orangutan (seven) relatively to the orangutan and macaca (three) (Figure 10). This suggests that different rates of protein evolution might have occurred in the evolution of the great ape ABAD/HSD10 proteins. Of the six positively selected sites in the branch separating human/chimpanzee from orangutan, three were localized in loop D (98, 106 and 108) and one in substrate-binding loop (214). It is also interesting to note that in two of the six positive selected sites (106 and 108), the changes occurring in the human/chimpanzee sequences were not seen in the other eutherian ABAD/HSD10 proteins, and in three sites (80, 119 and 214), amino acid changes occurred exclusively in the human/chimpanzee sequences with the other eutherian sequences containing the same amino acid.

## Discussion

Here, we present a comprehensive comparative genomic analysis of the HADH1 gene encoding ABAD/17β-HSD10 across 21 species. HADH2 gene revealed a substantial conserved organization across a large evolutionary distance: vertebrate and nematode HADH2 genes showed a six-exon/five-intron gene organization, but insects showed a reduced and varied number of exons (two to three). In general, HADH2 genes were less than 3.20 Kb long (Table 1; Figure 1), with the exception of zebrafish gene which was much larger (9.38 Kb). At nucleotide level, a notable characteristic of HADH2 genes was the extensive variation in GC3 content.

The reduced conservation in noncoding regions between human and the non-eutherian vertebrate orthologues, reinforce previous conclusions that in general, few noncoding functional sequences remain conserved for large evolutionary distances [33]. Several studies [34-36] have shown that the conservation in non-coding regions between human and distant vertebrate species (e.g., fishes) is restricted to a subset of genes that are involved in pivotal biological processes such as development and transcription regulation. These genes display a high density of conserved elements in their introns and intergenic regions, which is related with the need to preserve complex and crucial regulatory mechanisms in basic vertebrate development [36,37]. A recent broad study [38] about intron conserved elements between human and various vertebrates, including chicken and fishes, reported that multispecies conserved noncoding sequences distribution is not uniform across human introns. Indeed, the longer introns of the genes involved in development and transcription regulation showed a tendency to accumulate conserved sequences, while the majority of relatively short introns (< 9 Kb) displayed none or few conserved elements [38]. In view of the limited conservation between human and distantly vertebrates, it has been assumed that for the majority of human genes, comparisons between multiple, moderately related species might represent a better strategy to search for potential regulatory elements [39], although cautious is required as the high degree of similarity might also reflect low substitutions rates of evolution. It is possible that some of the HADH2 conserved noncoding sequences, identified in the comparisons between human and the five moderately related eutherian mammals might indeed represent regulatory elements, and thus be good candidates for functional experimental studies. For instance, the significant sequence similarity throughout the upstream regions, namely in the region about 1 kb of the transcription start site, may suggest the presence of regulatory elements, likely involved in transcription. The first part of intron 1 was found to be highly conserved among eutherian mammals and interestingly, it was the highest conserved intronic region between Tetraodon and Fugu. Given that intron-associated regulatory elements on genes tend to be localized preferentially in intron 1 [40], such pattern of conservation in eutherian mammals and in pufferfishes genes suggests that HADH2 intron 1 may potentially contain a regulatory element.

Previous studies [41,42] reported an increase in both exon and intron conservation in the regions flanking conserved alternative splice sites. The conserved alternative splicing event between human and dog HADH2 genes raises the possibility that some of the conserved intronic regions, particularly in the introns flanking exon 5 (intron 4 and 5), might reflect the presence of splicing regulatory elements subject to purifying selection. In addition, the high conservation found in the exons 5 and 6 likely reflect their importance in coding for residues of the substrate binding cleft, but it might also be related with the presence of conserved exonic splicing regulatory elements. The conservation of human and dog HADH2 alternative donor splice sites in other eutherian mammals, suggests they can potentially also express an identical alternative splicing variant. However, as previously noted [43,44], conservation of a splice site is not enough to predict the existence of a variant. Thus, the evaluation of identical alternative splice variants in the other mammals, including the one identified solely in dog, will help to further elucidate the potential functional importance of the conservation of intronic elements in eutherian HADH2 genes.

Curiously, the two identified alternative splicing events interfere with the enzyme C-terminal, an important functional region. In the dog variant 3 (169 amino acid), the last 92 amino acids are replaced by seven new ones. As this leads to the loss of two residues of the catalytic triad and of the substrate binding loop residues, likely, the dog variant 3 is non-functional. The production of non-functional transcripts is not uncommon, with many genes using that as a mechanism to control the mRNA expression levels [45,46]. In human and dog variant 2, the loss of strand β_F_, which is one of the seven strands that compose the core β-sheet of the ABAD/HSD10 Rossmann fold dinucleotide-binding motif, may have both structural and functional consequences, as this strand is adjacent to the region containing the substrate binding loop and is also involved in the substrate binding [2]. However, the fact that the substrate binding cleft residues are retained and the existence of a shared variant between human and dog suggest that it might be functional. The assessment of the alternative splice variants functionality, as well as the mechanisms regulating splice sites selection, and of the regional expression levels, will be fundamental to determine the implications of the alternative splicing in the physiological and pathophysiological functions of ABAD/HSD10. Of special importance is how ABAD/HSD10 alternative splice variants behave in an Aβ-rich environment? In this respect, since the human variant 2 has a normal N-terminal region, which is responsible for the interaction with Aβ [12], it may still retain the ability to interact with Aβ. Moreover, the human variant 2 is supported by an mRNA sequence (BC008708) derived from neuroblastoma cell lines, suggesting that it may be expressed in the human brain.

At protein level, ABAD/HSD10 showed a high similarity across the different species, namely in regions comprehending the substrate binding cleft and the subunit association, clearly suggesting that ABAD/HSD10 maintained a substantial structural and functional conservation across a very large evolutionary distance. Indeed, previous studies have demonstrated that the rat and fruitfly orthologues exhibit enzymatic activities similar to those of the human enzyme [2,47], suggesting that ABAD/HSD10 might have important functions both in vertebrates and invertebrates. However, despite being recognized that the broad substrate specificity of ABAD/HSD10 enables it to participate in several metabolic pathways, the physiological properties of the enzyme are not yet completely understood. In mammals, ABAD/HSD10 was suggested to have an important role in metabolism of sex steroid hormones [10]. It was reported to be expressed in the Leydig cells of testes from various mammals and the differentiation-dependent expression of ABAD/HSD10 in rodent testes suggested that this enzyme might contribute to protecting Leydig cells from the effects of estrogens [10]. The great importance of fruitfly ABAD/HSD10 (termed scully) was demonstrated by the mutational inactivation of the enzyme, which induced a lethal phenotype during embryonic and pupal development, with mutants displaying non-functional gonads, lipid accumulation and aberrant mitochondria [48]. Human ABAD/HSD10 deficiency causes a disorder in which the isoleucine degradation is impaired [6]. Patients with this deficiency show severe neurological abnormalities, including psychomotor retardation and progressive infantile neurodegeneration. A beneficial role in the cellular response to metabolic stress was attributed to the ABAD/HSD10 enzyme, due to its ability to utilize the ketone body β-hydroxybutyrate as a substrate [49]. ABAD/HSD10 may also be important in the stabilization of mitochondrial function [14] and in the maintenance of normal functions of GABAergic neurons [11].

Despite the high degree of conservation among eutherian ABAD/HSD10 proteins, suggesting strong purifying selection pressures, we investigated signatures of positive selection by using both a gene-level and protein-level approaches. The failure of the gene-level approach in providing significant evidence of molecular adaptation across eutherian mammal orthologues, probably reflect the known lack of power of the used LRTs in detect positive selection when divergence between sequences in the data set is low [50]. By contrast, the significant evidence of positive selection provided by the protein level approach indicates its ability to identify molecular adaptation even when proteins are highly conserved [51]. An interesting finding, was the detection of positive selection in important functional regions, in particular in the lineage separating human/chimpanzee from orangutan, which, contrasting with other eutherian mammal branches, accumulated a higher number of nonsynonymous than synonymous substitutions. Of the seven residues differing between human and orangutan, the protein-level approach detected six to be under molecular adaptation, four of which localized in particularly important functional regions. Specifically, three sites were localized in the loop D (site 98 in a region belonging to the substrate binding cleft and sites 106 and 108 in insertion 1 region) and a fourth site (214) was localized in the substrate binding loop. The previous sites 98 and 108, plus three additional sites (95, 102, 103) localized in loop D and site 202 belonging to the substrate binding loop were also identified to be under positive selection in other eutherian mammal branches. The potential functional meaning of the positive selection in eutherian mammal ABAD/HSD10 proteins, particularly in the regions belonging to the substrate binding cleft is, however, difficult to ascertain given the enzyme multiple substrate specificities and participation in various metabolic pathways. As stated behind, ABAD/HSD10 seems to have maintained a considerable structural and functional conservation across a very large evolutionary distance. However, it is important to say that although the identification of positively selected amino acid sites do not necessarily prove that such amino acid replacements modify the protein function, their occurrence in functional important sites provide a strong evidence for further functional experimental analyses. In this respect, particularly appealing is the evidence of positive selection in loop D, given its involvement in Aβ binding. Recently, the determination of the crystal structure of ABAD/HSD10 bound to Aβ and mutational studies on loop D furnished strong evidence for this loop functions as the binding site for Aβ [12].

## Conclusion

The sequencing of various genomes provided the opportunity to study the molecular evolution of the HADH2 gene encoding ABAD/HSD10. Our study revealed that HADH2 genes maintained a very similar organization and substantial conservation at amino acid level over more than one billion years. The identification of a conserved alternative splicing event between human and dog and highly conserved noncoding regions among eutherian mammals may provide a framework for further investigation of HADH2 gene regulation. The evidence of positive selection across eutherian mammal ABAD/HSD10 proteins may be of importance for more applied biomedical research on the enzyme function and its implication in the Alzheimer's disease.

## Methods

### Database search of HADH2 gene sequences

The sequences of the HADH2 gene encoding ABAD/HSD10 protein (synonymous names include SCHAD, ERAB, MHBD, and *scully *for the Drosophila orthologue) were retrieved from Ensembl [52], NCBI [53] and TIGR [54] databases (Table 1). Gene sequences were identified either by TBLASTN searches within the various species genome sequence projects using known ABAD/HSD10 amino acid sequences as queries or manually reconstructed from whole genome shotgun (WGS) traces through MEGABLAST searches. For some species, the HADH2 genes retrieved were only partially sequenced (see figure 1). Additionally, BLAST searches were performed against species specific mRNA reference sequence (RefSeq) databases at NCBI to detect alternative splicing variants.

### Sequence conservation

The amount and composition of repetitive elements was investigated using RepeatMasker [55], CENSOR [56] and Tandem Repeats Finder v.3.01 [57]. After removing repetitive motifs, Pip software analysis [58] was used to align and identify patterns of sequence conservation across vertebrates, fishes and nematodes HADH2 genes. The 2 kb region upstream the HADH2 genes was included in the analysis, excepting for cat and orangutan genes, where only a smaller portion was available (1 kb for cat and 200 bp for orangutan). Protein sequences were aligned with CLUSTALW [59]. Sliding window percent amino acid identity analyses (excluding indels) were conducted using Swaap 1.0.2 [60].

### Phylogenetic analyses

HADH2 sequences were investigated for variation in base composition (or compositional bias), mutational saturation, and gene conversion, which are events known to disturb phylogenetic reconstructions. The chi-square test of homogeneity implemented in TREE-PUZZLE v5.2 [61] was used to evaluate variation in base composition at each codon position. GENECONV v1.81 [62] was employed, using the default settings, to detect recombination/gene conversion events in the data set. To test for mutational saturation, we plotted the number of transitions and transversions from first, second, and third-codon positions against the pairwise genetic distances. The SYM+G+I model was identified with Modeltest v3.06 [63] as the best evolutionary model fitting the data. Transitions and transversions for all pairwise sequence comparisons were calculated using MEGA v3.1 [64], whereas genetic distances were calculated in PAUP v.4.0 b10 [65]. In the absence of mutational saturation, genetic distances and nucleotide substitutions give a linear relationship. Conversely, in case of nucleotide saturation, genetic distances are larger than substitutions [66,67].

The phylogenetic relationships among HADH2 sequences from different species were determined using Maximum-likelihood (ML) and Bayesian methods, implemented in PAUP v.4.0 b10 [65] and MRBAYES v3.1 [68], respectively. The ML tree was reconstructed through a heuristic search with ten random additions of taxa and tree bisection-reconnection (TBR) branch swapping algorithm. Bootstrap support (BS) values were estimated with 100 replicates. In the Bayesian analysis, four markov chains were run for 500,000 generations with burn-in values of 2,500 generations and trees being sampled every 100 generations. Bayesian posterior probabilities (BPP) were used to evaluate branch support. Both trees were rooted using *C. elegans *and *C. briggsae *sequences as outgroups.

### Detection of positive selection

Positive selection analyses were restricted to eutherian mammals to avoid violations in the evolutionary assumptions, i.e. absence of nucleotide saturation and base compositional bias, which requires closely related sequences (see Table 1 and Figure 6). We used two strategies to identify positive selection: (i) a gene-level approach based on the ratio (ω) of nonsynonymous (*d*_*N*_) to synonymous (*d*_*S*_) substitutions rate (i.e., ω = *d*_N_/*d*_S_), and (ii) a protein-level approach which evaluates the physicochemical importance of amino acid changes on the protein structure. The unrooted eutherian mammals ML tree was used in the analyses.

The gene-level approach implemented in PAML v3.14 [69] uses likelihood ratio tests (LRT) to compare two nested models, a model that does not account for sites with ω > 1 (null model) and a model that does (positive selection model) [70]. We used two LRTs based on site specific models, which compare the null models M1a and M7 against the alternative models (positive selection models) M2a and M8, respectively. The posterior probability of a site being under positive selection was obtained using the Bayes Empirical Bayes (BEB) method implemented in PAML [71]. We also constructed two LRTs based on branch models, the first compares one ratio model (M0, assumes the same ω ratio for all branches) with the free-ratios model (allows an independent ω ratio for each branch) and the second compares model M0 with the two-ratio model (assumes a ω ratio for foreground branch different from that of background branch). The two-ratio model was applied in a specific branch (see results for details).

The protein-level approach implemented in TreeSAAP [72] measures the selective influences of 31 physicochemical properties across a phylogenetic tree following McClellan and McCracken method [73]. The program uses a gradient of categories to classify each property change from conservative to radical, and calculates a z-score which indicates the direction of selection. In our analysis, we were interested in detecting positive-destabilizing selection as this results in radical structural or functional shifts in local regions of the protein, thus, being unambiguously correlated with molecular adaptation. An amino acid property is said to be affected by positive-destabilizing selection when the frequency of changes in radical magnitude categories exceeds the frequency(s) expected by chance, as indicated by positive z-scores.

VMD program [74] was used to map the positively selected amino acid sites on the crystal structure of human ABAD/HSD10 (PDB 1U7T) [15].

## Authors' contributions

ATM performed all sequence and phylogenetic analysis, comparative genomics, and drafted the manuscript, AA participated in the genetic analyses, design, drafting and co-ordination of the study, PAF and MJR participated in the drafting and coordination of the study. All authors read and approved the final manuscript.

## Supplementary Material

Additional File 1 **Complex and simple repeats present in HADH2 genes**. List of complex and simple repeats identified in the HADH2 genes analysed, by using the RepeatMasker, CENSOR and Tandem Repeats Finder programs. Click here for file

Additional File 2 **Pairwise amino acid sequence identity of ABAD/HSD10 orthologues**. Click here for file
