# Supplementary material for: Comparative evolutionary genomics of the HADH2 gene encoding Aβ-binding alcohol dehydrogenase/17β-hydroxysteroid dehydrogenase type 10 (ABAD/HSD10)
Source: BMC Genomics. 2006 Aug 9;7:202. doi: 10.1186/1471-2164-7-202 (PMC1559703; doi:10.1186/1471-2164-7-202)
Supplement: Additional File 1 — Complex and simple repeats present in HADH2 genes. List of complex and simple repeats identified in the HADH2 genes analysed, by using the RepeatMasker, CENSOR and Tandem Repeats Finder programs. [file 1471-2164-7-202-S1.pdf]

Additional file 1: Complex and simple repeats present in HADH2 genes

Specie	Intronic location	Repeat class	Repeat name	Percentage of intronic sequence (%)
human	intron 1	simple	(TAGA)n	13.42
	intron 2	LINE<br>SINE	L3<br>AluSx (2)	55.39
chimpanzee	intron 1	simple	(TAGA)n	16.36
	intron 2	LINE<br>SINE	L3<br>AluSx (2)	55.16
orangutan	intron 1	simple	(TAGA)n	12.96
	intron 2	LINE<br>SINE	L3<br>AluSx, AluSq	55.14
rhesus monkey	intron 1	simple	(TAGA)n	23.09
	intron 2	LINE<br>SINE<br>simple	L3<br>AluSx (2)<br>(CAGGG)n	53.83
rat	intron 2	SINE	RSINE1, BC1MM	24.34
mouse	intron 2	SINE<br>simple	ID4_, RSINE1<br>(G)n, (CA)n	30.72
cat	intron 5	SINE	SINEC_Fc2	48.50
opossum	intron 5	SINE<br>simple	MAR1, MIRb<br>(CCCCCA)n, (TTGGGTTCCAGT)n	53.80
western clawed frog	intron 3	unknown<br>simple	TE_ORF_98<br>(CACAG)n	21.43
zebrafish	intron 2	unknown<br>DNA transposon<br>low complexity<br>simple	Dr000372, Dr000461, Dr000462, Dr000415, Dr000149, Dr000194<br>TDR18, HATN13_DR, HATN16_DR (2), ANGEL<br>AT_rich (2)<br>(TA)n	66.29
	intron 3	low complexity	AT_rich (3)	9.83
	intron 4	DNA transposon<br>low complexity	ANGEL<br>AT_rich	9.90
honeybee	intron 1	low complexity	AT_rich	97.77
	intron 2	low complexity<br>simple	AT_rich (2)<br>(TTA)n, (TA)n, (TTTA)n	87.84
C. elegans	intron 2	DNA transposon	CELE1, CELE2 (2)	77.40
	intron 4	DNA transposon	CELE2	51.03

NOTE.— In the repeat name columm, for repeats found more than once in the same intron, the number of times is indicated in parenthesis
