# Supplementary material for: Comparative evolutionary genomics of the HADH2 gene encoding Aβ-binding alcohol dehydrogenase/17β-hydroxysteroid dehydrogenase type 10 (ABAD/HSD10)
Source: BMC Genomics. 2006 Aug 9;7:202. doi: 10.1186/1471-2164-7-202 (PMC1559703; doi:10.1186/1471-2164-7-202)
Supplement: Additional File 2 — Pairwise amino acid sequence identity of ABAD/HSD10 orthologues. [file 1471-2164-7-202-S2.pdf]

Additional file 2: Pairwise protein sequence identity of ABAD/HSD10 orthologues

	human	chimpanzee	orangutan	r. monkey	cow	pig	dog	cat	mouse	rat	opossum	w. c. frog	a. c. frog	zebrafish	puffer fish	fugu fish	fruitfly	mosquito	honeybee	*C. elegans*	*C. briggsae*
human	1.00																				
chimpanzee	1.00	1.00																			
orangutan	0.97	0.97	1.00																		
r. monkey	0.97	0.97	0.99	1.00																	
cow	0.92	0.92	0.93	0.93	1.00																
pig	0.91	0.91	0.92	0.91	0.94	1.00															
dog	0.92	0.92	0.93	0.93	0.93	0.91	1.00														
cat	0.93	0.93	0.94	0.94	0.95	0.92	0.96	1.00													
mouse	0.88	0.88	0.89	0.89	0.90	0.87	0.89	0.89	1.00												
rat	0.88	0.88	0.89	0.90	0.90	0.87	0.89	0.89	0.93	1.00											
opossum	0.73	0.73	0.74	0.74	0.74	0.73	0.74	0.74	0.73	0.72	1.00										
w. c. frog	0.77	0.77	0.77	0.77	0.78	0.77	0.76	0.77	0.77	0.75	0.74	1.00									
a. c. frog	0.76	0.76	0.76	0.76	0.77	0.75	0.75	0.76	0.75	0.73	0.74	0.96	1.00								
zebrafish	0.74	0.74	0.74	0.74	0.75	0.74	0.75	0.75	0.73	0.72	0.74	0.81	0.81	1.00							
Tetraodon	0.73	0.73	0.74	0.73	0.74	0.73	0.74	0.75	0.74	0.73	0.73	0.79	0.78	0.86	1.00						
Fugu	0.75	0.75	0.76	0.75	0.77	0.75	0.76	0.77	0.74	0.73	0.75	0.81	0.80	0.90	0.95	1.00					
fruitfly	0.67	0.67	0.68	0.68	0.68	0.66	0.69	0.68	0.66	0.65	0.64	0.69	0.68	0.67	0.65	0.67	1.00				
mosquito	0.67	0.67	0.67	0.67	0.66	0.65	0.67	0.67	0.65	0.63	0.62	0.7	0.69	0.66	0.63	0.65	0.82	1.00			
honeybee	0.60	0.60	0.61	0.61	0.62	0.60	0.60	0.61	0.60	0.59	0.60	0.63	0.63	0.60	0.61	0.61	0.63	0.65	1.00		
C. elegans	0.59	0.59	0.59	0.61	0.59	0.57	0.57	0.57	0.59	0.59	0.53	0.57	0.57	0.57	0.57	0.57	0.57	0.56	0.55	1.00	
C. briggsae	0.59	0.59	0.58	0.58	0.59	0.57	0.58	0.58	0.59	0.58	0.52	0.56	0.57	0.55	0.56	0.55	0.56	0.55	0.54	0.95	1.00
